# Chemistry in the Practice of Dentistry

**Published:** 1882-06

**Authors:** H. Lee Davis


### THE DENTAL REGISTER.

VOL. XXXVI.] JUNE, 1882. [No. 6.

### CHEMISTRY IN THE PRACTICE OF DENTISTRY.

BY H. LEE DAVIS, D.D.S.

As anatomy is to surgery, so is chemistry to dentistry, especially to operative dentistry, of which it may, in some sense, be regarded as the base. When we look at the "why" of each step in operative dentistry we find that it is based on chemical principles, and when we first begin to examine a patient's mouth, the defects we find are the results of chemical compositions and decompositions. So we can already see the need of a knowledge of chemistry in the practice of dentistry. A knowledge of chemistry enables us to know that the teeth, saliva, mucus, salivary calculus, etc., are chemical compositions, while decay, abrasion, ulcerations, etc., are decompositions, the effects of certain compositions, caused by the saliva, particles of food and other elements foreign to the mouth, uniting to form definite chemical compounds of which we will take more notice as we proceed.

First let us look at the constituents of saliva, which constantly bathes the mouth and organs of mastication.

The importance of a thorough knowledge of the fluids of the mouth can hardly be overrated, since they mingle themselves with the food, and exert a powerful influence over digestion, and as digestion varies the fluids of the mouth will vary, while the reaction of normal mucus is acid and of saliva alkaline, yet in health, when combined, the reaction is neutral, but if either is in excess it is alkali, or acid as the case may be, but as digestion changes these fluids may change, and become excessive in alkalinity or acidity, in which case they will act more readily on the teeth, although an article has lately been published from a practicing dentist in which he says that he has made an analysis of saliva in a number of cases where there was much and rapid decay of the teeth, and he found that the reaction was not acid, but nearly neutral, this again shows us the need of chemistry and opens up a field of work for the dental chemist.

In healthy saliva, according to an analysis by Dr. Wright, we find water, ptyalin, fatty acid, chlorides of sodium and potassium, albumen with soda, phosphate of lime, albuminate of soda, lactates of potash and soda, sulphocyanids of potassium, soda and mucus, a knowledge of these chemical compounds enables us to understand how, in disease, this secretion may be changed in character, and by uniting with particles of food in the mouth, cause decomposition and setting free the elements that go to form some of the most destructive agents in the mouth, these combine together, according to chemical laws, and form acids which destroy the teeth, which we shall notice more fully in the causes of decay.

Saliva has the power of modifying, and, to a certain extent, of digesting vegetable and animal substances, but it has a more powerful action upon vegetable than upon animal matter. " The healthy digestive action of saliva is always attended with the evolution of lactic acid (Dr. Wright). This acid, together with acetic, hydrochloric, oxalic and uric, are those with which the saliva is most commonly contaminated, and we should have a knowledge of all these and more, as they corrode the teeth with great rapidity; aside from this the saliva is the chief agent in the deposit of salivary calculus or tartar. Salivary calculus or tartar is composed of phosphate of lime, carbonate of lime, animal matter, mucus and water. The calcareous material is brought into the mouth in a state of solution in the saliva, being secreted from the blood, while the fats and other animal matters are accumulated from the food, this is precipitated in the mouth and collects on the teeth, the greatest amount is found where the saliva is most abundant.

Next let us look at the constituents of the different parts of the teeth.

First, that which forms the greatest part of the tooth substance, and which is most effected by decay. The dential dentine is formed of

| | |
|---|---|
| Organic matter | 27.6 |
| Fat | 0.4 |
| Phosphate of calcium | 66.72 |
| Carbonate of calcium | 3.36 |
| Phosphate of magnesium | 1.08 |
| Other salts | 0.83 |

Thus we see it is closely related to bone in its chemical constituents, although it is harder and more dense. It is not necessary to dwell on these salts, constituting dentine, as they are all common to the chemist, and their composition will be familiar to all dentists who understand chemistry, if not, all the more reason we should make it a study, so as to understand the material we are working; as much, or more so, as the stone-cutter should understand the stone he is cutting, or the iron-worker the metal he is using; if either of these latter should spoil a piece of material, it can be supplied by another equally as good, but if the dentist should spoil a tooth he has done an irreparable injury.

Let us next look at enamel, the hardest substance in the body, and found only on the teeth, where it serves as a shield and protection to the softer dentine, so that the tooth will better stand the use to which it is subjected.

Enamel is composed of phosphate of lime 85.3, fluate of lime 3.2, carbonate of lime 8, phosphate of magnesia 1.5, soda and muriate of soda 1, animal matter and water 1. These proportions are not always the same they vary in the enamel of the teeth in different persons, some being hard and flint like, others being less dense, and sometimes so soft as to readily wear away and leave the dentine exposed. Enamel, as a general thing, does not succumb to the ravages of decay as easily as dentine, as we will find that only along the margins of the fissures where the enamel has failed to unite and decay has set in, in the dentine, will the enamel be decayed, and again we will find that the enamel alone has been the subject of decay, leaving the dentine intact as in denuding of the teeth. This is owing to the difference in its chemical compositions.

Next the cementum, it is more nearly allied to the formation of bone than the other parts of the tooth, it is not so dense and does not need to be, owing to its protected situation by the alveolus and gums, and hence it is not so liable t› decay as the others but does undergo the same changes when exposed.

Now that we have reviewed the composition of the saliva, dentine, enamel and cementum, let us see what changes may occur in these through chemical agents. We have seen that all these different parts are composed of definite chemical compounds, which are formed by definite chemical principles, by the union of two or more elements; hence they may be broken up by the addition of another element or elements which has a greater affinity for one or more of the elements composing the original compound, thus forming new compounds which destroy the identity of the old, thus breaking down the tissue and forming a pathological, or diseased condition of the parts.

As operative dentists, that diseased condition of the teeth that we have the most to do with is decay; so we will look into the causes of decay.

As we have said the first portion of the tooth that is subjected to the influence of decay producing agents is the enamel, but we sometimes find it very little affected, while the dentine beneath may be much decayed; this is owing to defects in the enamel which may be on account of its not uniting in the fissures of a tooth, or from cracks or checks in it, or pits upon its surface which form a lodging place for the decay producing agent, and as the enamel is not so likely to be affected, the agent passes through these places and attacks the dentine. This agent is generally an acid which breaks down the dentine.

The acids that effect the teeth in decay are the following: nitric, hydrochloric, sulphuric, acetic, lactic, malic, citric, etc.

To follow out the action of these acids would not only be a tedious task, but one that would be too long for a paper of this kind, and also unnecessary for the object of the paper. Hence we will only follow a few of the more important that figure most prominently in oral chemistry.

Oral chemistry treats of the different combinations formed in the mouth by the secretion, food, etc.

The food contains many substances, both of the hydro-carbon and nitrogenous classes, which, in the presence of warmth and moisture, will take on fermentation or putrefaction as the case may be. These processes, though closely allied, have each a distinct mode of action.

Fermentation has for its subjects the hydro-carbons and is generally dependent on the presence of a putrescing nitrogenous body. Its object is the re-arrangement of the atoms forming generally not more than two compounds.

Putrefaction takes place only in molecules containing nitrogen, the atoms of which are liberated to reunite according to their affinities forming, various compounds.

The mouth presents the conditions necessary to the first process, warmth and moisture, and not only ptyalin of the saliva, which is itself a ferment, but very often decomposing nitrogenous matter is present.

Glucose, from cane sugar, or starch, etc., with water and sugar of milk, readily ferments under these conditions and is formed into acetic or lactic acid, as the case may be.

In order to understand these chemical changes, we will have to look into the molecular construction of these and other substances as we take them up.

First glucose $C_6 H_{12} O_6$ contains all the atoms for the construction of acetic acid $C_2 H_4 O_2$ by a re-arrangement, but it is probably first converted into cellulose and then alcohol, as starchy substances undergoing fermentation is first changed into cellulose and this into alcohol and carbonic acid, thus $C_6 H_{12} O_6$ changes into $2 CO_2 + 2 C_2 H_6 O$, the alcohol is again broken up into aldehyde and water by the addition of an atom of O, and becomes $C_2 H_6 O + O = C_2 H_4 O + H_2 O$, then by another addition of O to the aldehyde it is converted into acetic acid thus: $C_2 H_4 O + O = C_2 H_4 O_2$, thus we have the formation of acetic acid in the mouth.

Lactic acid is brought about by the union of sugar of milk $C_{12} H_{22} O_{11}$ and an atom of $H_2 O$ (water) which going through the steps of fermentation finally forms lactic acid $C_3 H_6 O_3$.

In the putrefaction of animal tissue there are many complex primary chemical reactions, but the products of the secondary reactions are ammonia, sulphurretted hydrogen, hydrogen and nitrogen, besides the formations of fatty acids and glycerine. Here we find ammonia $NH_3$ and from this nitric acid $HNO_3$ is formed. Oxygen unites with $NH_3$ and we have $NH_3+O=H_2O+NH$ that is water with a fraction of N and H each, this takes up O enough to form $HNO_3$ nitric acid, and this acts very powerfully on the teeth at the moment of its formation and then disappears in the new compound.

This agent causes the white decay of the teeth, by uniting with the carbonate of lime which it decomposes and dissolves the phosphate of lime, sets carbonic acid free and forms nitrate of calcium, hence the equation $2\ HNO_3+CACO_3=CA(NO_3)_2+CO_2+H_2O$. After this decay has once begun and penetrated to the dentine an alkali may cause further decay by decomposing the animal matter of the tooth which forms about 28 per cent.

Sulphuric acid is formed in the mouth by the decomposition that is constantly going on there, the sulphur which is one of the constituents of the food and also of the mucus, will, when liberated, form with nascent hydrogen, sulphurretted hydrogen which may often be perceived on the breath, this decomposes in the presence of free O in the mouth and forms sulphuric oxide $SO_3$, this having a strong affinity for water will combine with it and form sulphuric acid, thus $SO_3+H_2O=H_2SO_4$. This acts on the carbonate of lime, forming sulphate of lime, and setting free $CO_2$ and $H_2O$, thus, $CACO_3+H_2SO_4=CASO_4+H_2O+CO_2$. This sulphate is insoluble in the saliva and helps to protect the tooth from further attacks. This acid may also help to arrest decay by de-hydrating the animal constituents of the tooth, as it takes away hydrogen and oxygen and leaves a black, dry, hard carbonized substance which protects the tooth from further decomposition. Hydrochloric acid is generally formed in the mouth from salt or chloride of sodium which is present in animal flesh and superadded when the flesh is cooked. This chloride of sodium is decomposed by the sulphuric acid into sulphate of soda and hydrochloric acid or by nascent hydrogen, which has a stronger affinity for chlorine than the sodium, and thus the H and CL uniting forms HCL, hydrochloric acid. Also this acid may be formed in the mouth by the electric action of fillings, as an amalgam and a gold filling coming in close proximiy, and thus setting up an electric current, which will decompose the salt and set the chlorine free, which owing to its great affinity for hydrogen will combine with it, and when this acid is once formed it will act as the corrosive liquid for the battery and thus promote the formation of the acid.

This acid does not decompose the calcium phosphate but holds it in solution, while the carbonate is decomposed into calcium chloride, $CO_2$ and $H_2O$, thus, $CACO_3 + 2\ HCL = CACL_2 + CO_2 + H_2O$.

This acid produces the well known brown decay, leaving the organic compounds in the cavity as debris.

Thus we have seen the effects of some of the more common acids in decay of the teeth, and we cannot help but see the advisability and necessity of a knowledge of chemistry as dentists, for these things come before us every day in our practice, and we should be as thoroughly acquainted with them as we are with our instruments, so that when we examine a patient's teeth, we can at least approximate the cause if not get at it directly.

Nor is this all, we should study and understand the chemical effects of all medicines used by us, and not be satisfied with a partial knowledge, as, knowing that we use carbolic acid for an escharotic, or arsenous acid to devitalize a pulp, or iodine to allay inflammation, etc., merely because they are used, but we should know the chemical effect of each, know what combinations they form and what changes they cause to take place in the tissues to which they are applied; as for carbolic acid it coagulates the albumen and fibrin, so, also, does tannic acid thus forming an insoluble compound that cannot be absorbed into the blood, also when tannic acid is taken into the stomach it combines with pepsine, and thus depriving it of its digestive power, retards digestion, but it can be changed into gallic acid by a union with water and thus taking up oxygen enough to form gallic acid which can be absorbed.

In the application of zinc chloride to exposed pulps, its chemical effects are to give up its chlorine which, owing to its great affinity for hydrogen, forms hydrochloric acid setting oxygen free, which tends to bleach the tooth and also unites with the zinc to form zinc oxide. This HCL formed, acts as an escharotic to the pulp, and if in sufficient quantity would attack the dentine.

